# Establishment of an electronic library in forensic psychiatric wards: a survey of its actual use by forensic inpatients

**DOI:** 10.1186/s12888-025-07632-3

**Published:** 2025-11-15

**Authors:** Koji Takeda, Teruyuki Nomura, Mio Suzuki, Sachiko Akita, Kiyoshi Takao, Akiko Tagami, Shin Yasuda, Taro Matsuda, Hiroyuki Wada, Ikuko Arakawa, Takao Suzuki, Emiko Yanagi, Yuka Ichihashi, Kazuhisa Tateyama, Kaori Matsumi, Ichiro Hanada, Ryota Hashimoto, Naotsugu Hirabayashi

**Affiliations:** 1 Department of Forensic Psychiatry, National Center Hospital, National Center of Neurology and Psychiatry, 4-1-1 Ogawa-Higashi, Kodaira, Tokyo 187-8551 Japan; 2 National Hospital Organization Saigata Medical Center, Jōetsu, Japan; 3 Niigata University of Health and Welfare, 1398 Shimami-cho, Kita-ku, Niigata 950-3198 Niigata, Japan; 4 National Hospital Organization Komoro Kogen Hospital, Komoro, Japan; 5 National Hospital Organization Ryukyu Hospital, Kunigami, Japan; 6 Shimane Psychiatric Medical Center, Izumo, Japan; 7 Okayama Psychiatric Medical Center, Okayama, Japan; 8 Kanagawa Psychiatric Center, Yokohama, Japan; 9 Osaka Psychiatric Medical Center, Hirakata, Japan; 10 National Hospital Organization Hanamaki Hospital, Hanamaki, Japan; 11 Department of Psychiatry, Tokyo Metropolitan Matsuzawa Hospital, Setagaya, Japan; 12 Department of Clinical Psychology, National Center Hospital, National Center of Neurology and Psychiatry, Kodaira, Japan; 13 Department of Nursing, National Center Hospital, National Center of Neurology and Psychiatry, Kodaira, Japan; 14 Rehabilitation Medicine, National Hospital Organization Kurihama Medical and Addiction Center, Yokosuka, Japan; 15 TRC Library Service Inc., Bunkyō, Japan; 16 Library Business Promotion Department Cultural, Educational and Library Business Promotion Division, Honto Business Center Publishing Innovation Operations, Dai Nippon Printing Co., Ltd, Shinjuku, Japan; 17 Department of Pathology of Mental Diseases, National Institute of Mental Health, National Center of Neurology and Psychiatry, Kodaira, Japan

**Keywords:** Forensic psychiatry, Electronic library, Information access

## Abstract

**Background:**

Similar to prison inmates, patients in forensic psychiatric wards may have restricted access to information. From a human rights perspective, improving information access is a desirable objective.

**Methods:**

A dedicated electronic library (e-library) was set up for use by forensic inpatients in Japanese forensic psychiatric wards. Forensic psychiatric inpatients who consented to participate in the study accessed the e-library with Information and Communication Technology devices. Demographic and psychotropic prescription information, e-library usage information, and the results of psychological tests were collected. Additionally, the relationship between behaviour associated with e-library use and the patients’ psychiatric symptoms were analysed.

**Results:**

The mean age of the 91 participants was 41.9. The total number of lending electronic books was 3429. The average and median number of logins were 48.3 and 20.0, respectively. The most common psychiatric diagnosis was schizophrenia spectrum disorders (89.0%). The ‘over 20 times login’ group had a significantly lower clozapine prescription rate and a higher full Intelligence Quotient score than the ‘less than 20 times login’ group. The group with higher logins also exhibited significantly higher scores in Bodily pain and Mental health scores on the Short Form-8 Health Survey.

**Conclusions:**

Physical and mental health may be positively associated with e-library use. The results suggest a demand for e-libraries among forensic psychiatric inpatients and have implications for policies about supplying e-libraries for forensic psychiatric wards.

**Clinical trial number:**

Not applicable.

## Background

Inmates in closed environments such as correctional facilities often have limited access to information. For example, within prisons, access to information is restricted for reasons such as facility and public safety and security [1]. However, such restrictions should be limited to the minimum level necessary to protect the right to freedom of expression [2]. Prisoners also have the right to access information [3], and providing prisoners with free access to appropriate information is important for their rehabilitation and reintegration into society [4].

Although forensic psychiatric wards vary globally, their primary role is to admit patients with mental illnesses who have committed criminal acts or are at high risk of doing so. In many countries, the length of hospital stay can be several years [5]. For example, in Japan, only offenders with mental illnesses who have committed serious crimes and are judged to have either a lack of criminal responsibility or reduced criminal responsibility due to mental disorder at the time of their crimes are admitted to forensic psychiatric wards [6]; the average length of stay is approximately three years [7]. Forensic psychiatric inpatients are treated like prison inmates in that strict controls are in place to prevent escape, their length of stay is long, and social reintegration and prevention of recidivism are important goals after discharge.

Public libraries are classic and important sources of information; however, prison inmates and forensic inpatients are not allowed to leave their institutional premises, or have limited opportunities to do so. Consequently, they do not have access to public libraries. Prison libraries play an important role in enabling prisoners to access information and reading materials [8]. A study of prisoners in Nigeria found that their information needs were diverse, with a particular need for legal support and health information [9]. Hussain et al. [10] reported that many prison libraries exist worldwide, and that the services they provide vary greatly. They state that ‘Prison libraries can open the door to rehabilitation, education and socialization for prisoners.’ [10].

Rehabilitation, education and socialisation are also critical issues for forensic psychiatric inpatients. However, to the best of the author’s knowledge, there are no reports on libraries in forensic psychiatric wards. Japan has 35 forensic psychiatric wards, with the number of beds ranging from five to 66 [11]. Although many Japanese forensic psychiatric wards have books for patients, the number is limited, and, often, the content is skewed toward comic books or magazines donated by former inpatients or ward staff.

In recent years, the main source of information has shifted to the Internet for the general public. However, in correctional facilities, Internet use is limited [10]. In prisons, Internet access is strictly limited not only for security reasons—such as preventing escapes or illegal activities—but also due to concerns about public backlash over the perception that internet access is luxury for inmates [12]. However, inadequate access to digital technology among individuals in the criminal justice system may hinder rehabilitation and increase the risk of reoffending [12]. Internet access via ICT devices in prisons can provide benefits such as video conferencing and educational platforms, including the acquisition of digital skills necessary for reintegration into society [13]. Therefore, it is desirable to promote internet access via ICT devices under appropriate regulations, aiming to balance safety and rehabilitation [13].

Supervised use of the Internet is likely to be common in forensic psychiatric units [14, 15], although not to the same extent as in correctional facilities. In 12 of 35 forensic psychiatric wards in Japan, patients were able to use the Internet under staff supervision, although they are not permitted to use smartphones in most wards [16]. In addition to providing the same aspects of facility security as correctional facilities, psychiatric wards must be careful to provide reliable information that does not interfere with treatment, prevent social loss owing to delusions or manic states that result in inappropriate emails or social media posts, and protect the personal information of patients and staff [15]. Furthermore, some forensic inpatients have had their names made public on the Internet because of news reports of their index offences (such as murder, rape or robbery). Therefore, forensic psychiatric wards must be careful about the possibility of negative effects on patients’ mental state resulting from viewing news reports on the Internet.

Electronic libraries (e-libraries) provide access to services and collections of electronic resources through Internet services and do not require physical space to house books [17]. Their use in prisons has been increasing worldwide. JSTOR is an e-library with more than 12 million materials and is used in more than 1,000 prisons [18]. E-libraries have become increasingly popular in Japan, with 32.7% of municipalities having them as of January 2025 [19]. However, e-libraries have not yet been introduced in Japanese prisons. Furthermore, to the best of our knowledge, no e-libraries have been introduced in forensic psychiatric wards worldwide. Forensic psychiatric wards have limited space; however, e-libraries do not require physical space. Furthermore, with e-libraries, staff can select books, ensuring that only reliable information is provided to patients.

Therefore, introducing e-libraries in forensic psychiatric wards would make it possible to improve patients’ access to information safely without reducing the physical space of the wards. Under these circumstances, we planned to create an e-library that could be used by Japanese forensic psychiatric wards to improve access to information for inpatients.

This study aimed to establish an e-library for patients in Japanese forensic psychiatric wards. Furthermore, it investigated the actual usage of the e-library by forensic psychiatric inpatients and the factors associated with its use. Additionally, it examined the relationship the between e-library use and psychiatric symptoms by conducting psychological evaluations at the start of e–library use and periodically thereafter.

## Methods

This study was conducted with designated forensic inpatient facilities, Dai Nippon Printing Co., Ltd. (DNP), and TRC Library Service Inc. (TRC). This study used LibrariE and TRC-DL, an e-library platform provided by DNP and TRC. This study was a prospective cohort study. We established an e-library specifically for forensic psychiatric inpatients. Study participants were given individual e-library IDs and used the e-library according to their interests. They also answered psychological tests at the time of participation and again after 4 weeks, 12 weeks, and 26 weeks. Researchers collected the basic demographic data of the study participants as well as data on their use of the e-library.

### Electronic library

LibrariE & TRC-DL is the most commonly used cloud-based e-library services in Japan. While the platform was originally intended for public or school libraries, DNP and TRC provided it free of charge, specifically for this study. However, the cost of the contents for the designated facilities’ e-librariy (electronic books: e-books) was borne by the National Center of Neurology and Psychiatry, using a grant from the Mitsubishi Foundation (Mitsubishi Zaidan). The line-up of the e-library (e-books) was based on the library collection ratio of junior high and high schools in Japan. In addition, we included a slightly larger number of books on psychiatry and psychology, as well as on social functions such as cooking and housework. We also included self-produced educational material related to forensic psychiatry. Moreover, we selected books requested by the participants based on their questionnaires. The number of collected e-books changed over time as many of the books had 2-year licence contracts. In addition to the e-books we selected and purchased based on the above criteria, 500 copyright-free older books (selected from Aozora Bunko [20]) were also available for viewing through the e-library. Most of the e-books in Aozora Bunko are classified in Category 9, thus Category 9 has more books than other categories. The maximum number of e-books was 1478 and the minimum number was 772 (excluding self-produced educational materials).

**Fig. 1:**
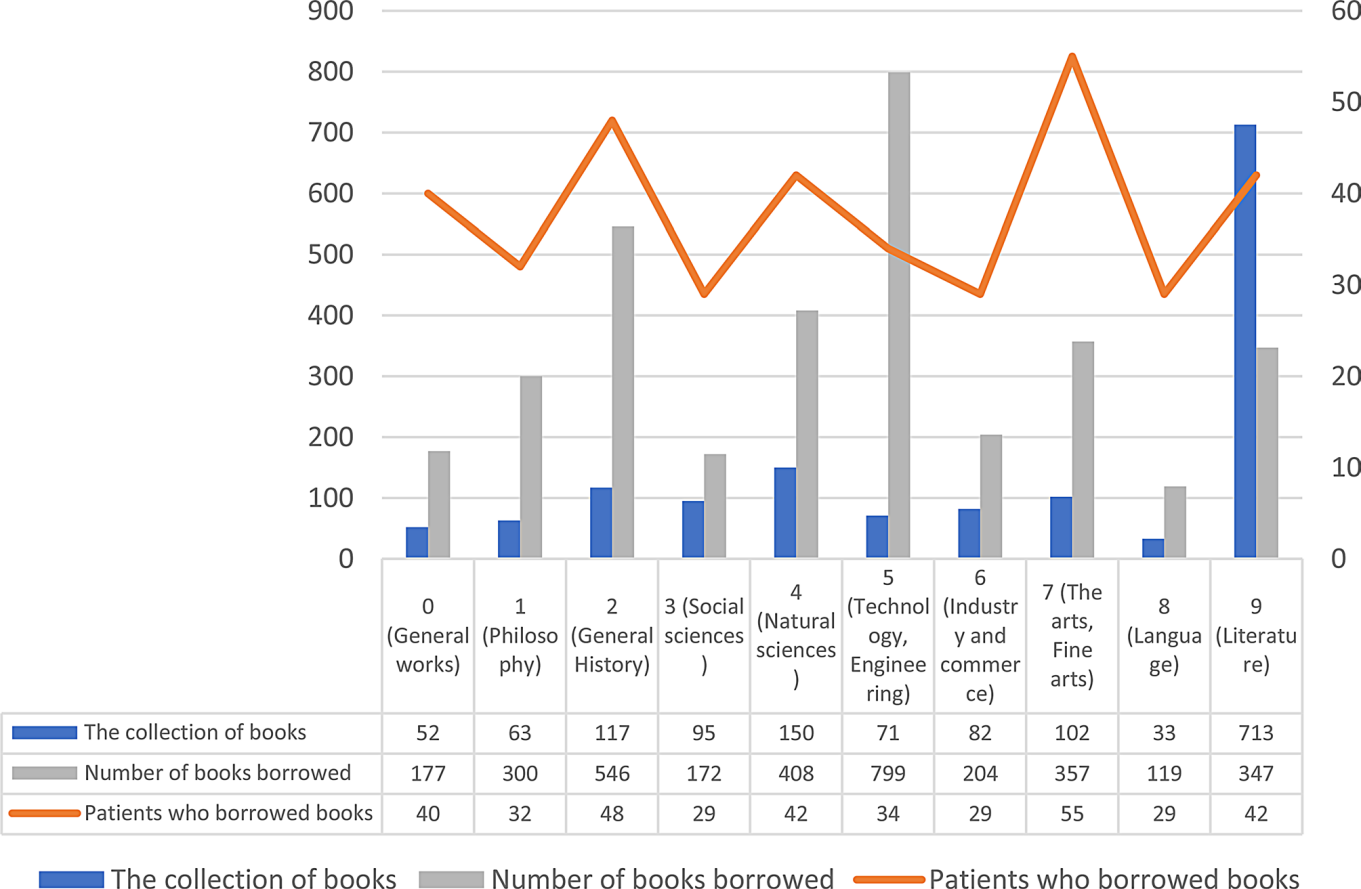
The electric library’s collection of e-books and borrowing status (classified by NDC code version 9)

Figure 1 shows a collection of the e-library. Nippon Decimal Classification (NDC) codes have been used to classify the types of books in Japanese public libraries [21].

### Reading environment

Participants used Information and Communication Technology (ICT) devices, such as digital tablets and PCs, to access the e-library. The type and number of ICT devices available for browsing the e-library and the browsing hours and available locations differed at each facility. In some facilities, participants were able to browse the e-library on tablets in their own rooms, whereas in others they were able to browse on PCs installed in communal areas.

Participants logged into the e-library using their ID and password each time. After logging in, they could borrow and browse e-books according to their interest. Participants could return the e-books themselves, but if they forgot to do so, the e-books were automatically returned after the lending period (7 days) had expired. They could borrow up to three e-books simultaneously.

### Security environment of ICT devices

As participants used the e-library with ICT devices, without the supervision of ward staff, researchers at each facility installed mobile device management, parental control, web filtering, and other software to restrict the use of other applications and website viewing. This block prevented the viewing of information that was unreliable, offensive to public order and morals, or detrimental to patients’ psychiatric symptoms; it also prevented the leakage of personal and ward security information via the Internet. The details of the security of ICT devices were left to the discretion of the wards and therefore varied.

### Participants

Forensic psychiatric inpatients who provided written informed consent participated in this study. The research was conducted between 1 February 2021 and 31 March 2024. Of the 35 facilities nationwide with forensic psychiatric wards, ten initially participated in the study. However, as one of the facilities was unable to create a suitable environment for the safe use of ICT equipment and could not start the research, the study was conducted at nine facilities.

### Demographic and psychotropic prescriptions information

The researchers collected the following information from the medical information: age, sex, psychiatric diagnosis based on the International Classification of Diseases, 10th edition (ICD-10), index offences, duration of hospitalisation, academic history, employment history, Wechsler Adult Intelligence Scale (WAIS-III or IV) score, and psychotropic prescriptions. The WAIS-III was published in 1997 [22], and the WAIS-IV was published in 2008 [23]. The Japanese versions of the WAIS-III and WAIS-IV were published in 2006 and 2018, respectively. We also surveyed participants regarding their use and possession of ICT devices, reading habits, and frequency of public library use, prior to hospitalisation.

### Use of e-library

We extracted data for participants using e-library, such as user ID, login, borrowing, and viewing books, from the statistical information of the e-library. The duration of e-library use was calculated as the number of days from the first e-library login date to the last login date for each patient.

### Psychological tests

Psychological tests were conducted to evaluate the association between e-library usage and psychiatric symptoms. The participants responded to self-reported psychological tests at the beginning of the study and at 4, 12, and 26 weeks after the start of the study. The psychological tests included the General Self-Efficacy Scale (GSES), Short Form-8 Health Survey (SF-8), Brief-Coping Orientation to Problems Experienced Inventory (Brief COPE), and Beck Cognitive Insight Scale (BCIS).

The GSES is a psychological test of general self-efficacy in various life situations; it consists of 16 items, and patients answer the items with Yes or No [24]. Standardised points were used for the statistical analysis of GSES in this study.

The SF-8 was published in 2001 [25]. The SF-8 evaluates health-related quality of life (HRQOL), which is a psychological test consisting of four levels of options and eight items [25]. The items were physical functioning (PF), role physical (RP), bodily pain (BP), general health (GH), vitality (VT), social functioning (SF), role emotional (RE), and mental health (MH). The physical component score (PCS), and mental component score (MCS) were calculated from the scores of those items. The SF-8™ Japanese version was used in this study [26].

The Coping Orientation to Problems Experienced (COPE) inventory was developed by Carver et al. [27]. It is a multidimensional coping inventory that assesses different ways in which people respond to stress. The Brief COPE was developed, based on the COPE, to reduce the time required to complete the questionnaire [28]. The Brief COPE consists of 28 items and 14 subgroups, which include self-distraction, active coping, denial, substance use, use of emotional support, use of instrumental support, behavioural disengagement, venting, positive reframing, planning, humour, acceptance, religion, and self-blame. We were provided with the Brief COPE Japanese version by Otsuka [29].

Beck et al. developed the BCIS in 2004 [30]. The BCIS measures cognitive insight of patients with schizophrenia [30]. It is a 15-item questionnaire which includes a nine-item self-reflectiveness subscale and a six-item self-certainty subscale. The BCIS composite index score is calculated by subtracting the self-certainty score from the self-reflectiveness score. The development of the Japanese version of the BCIS (BCIS-J) and its reliability and validity were reported by Uchida et al. [31]. We were provided with the BCIS-J questionnaire for this study by Kikuchi, the co-author of that report.

### Statistical analysis

**Fig. 2:**
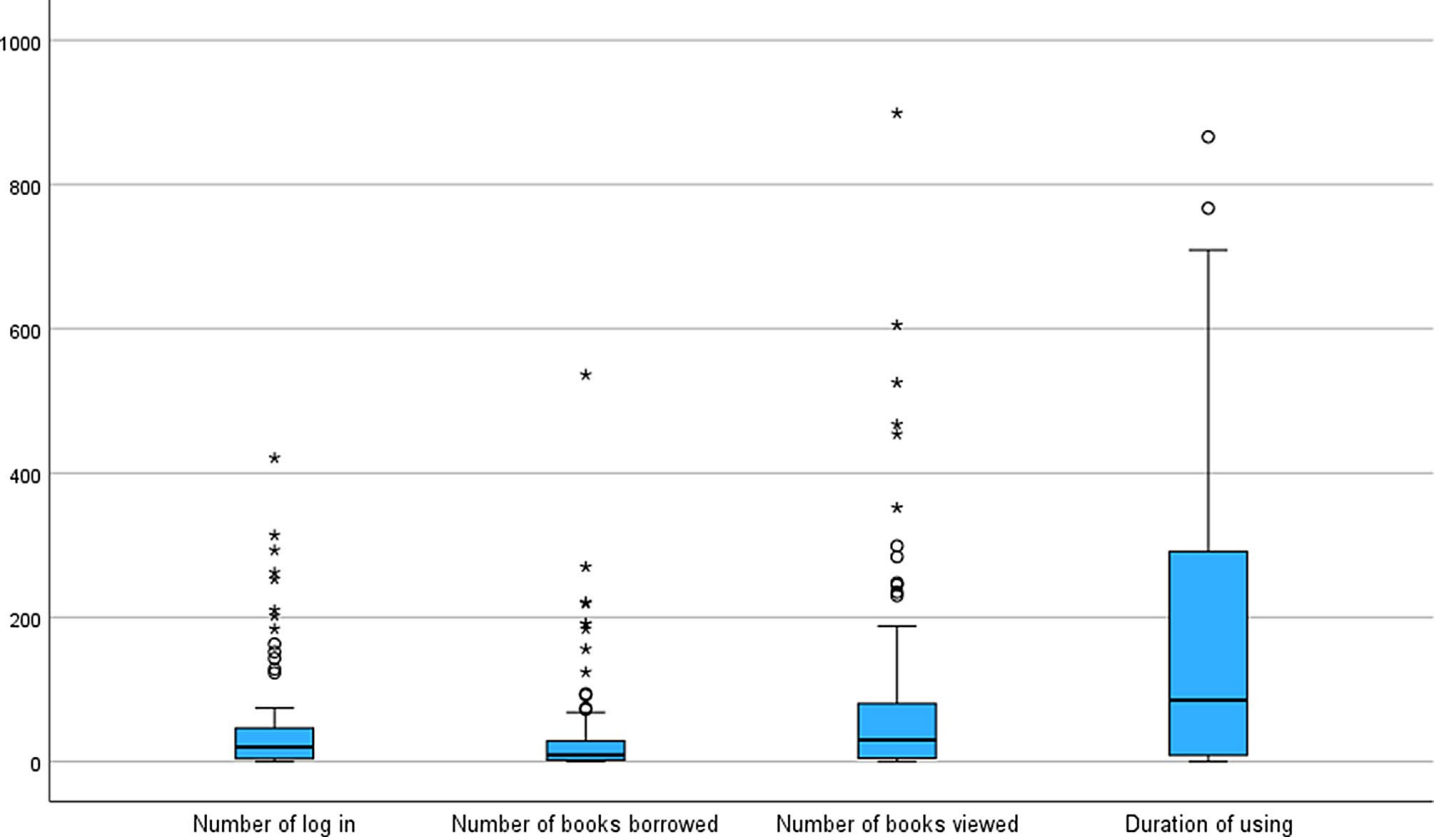
Distribution of the e-library usage, box-and-hide diagram

All statistical analyses were performed using SPSS Statistics (version 27.0; IBM, Armonk, NY, USA). The usage of the e-library (Fig. 2) consisted of non-parametric data with large individual differences. The number of logins was highly correlated with the number of books borrowed, number of books viewed, and the duration of e-library use (Appendix 1). Therefore, statistical analyses of demographic and psychotropic prescription information and results of psychological tests at the start of this study were conducted by dividing the participants into two groups based on the median number of logins (20 times). In these descriptions and exploratory analyses to reveal the association between each factor and e-library use, continuous variables were analysed using t-tests (parametric data) or Mann-Whitney U tests (non-parametric data), and categorical variables were analysed using chi-squared tests or Fisher’s exact tests.

We also analysed changes in the results of psychological tests. To evaluate the relationship between the use of an e-library and changes in psychiatric symptoms, we needed to analyse the participants who used the library frequently. Thus, we analysed changes in the results of psychological tests in participants who logged in more than 20 times using the Wilcoxon signed-rank test (Baseline vs. 4-week, 12-week, and 26-week). We selected list-wise deletion; thus, only participants who submitted all psychological test data were included. The Bonferroni method was used for *p*-value adjustment (*p*-value multiplied by three).

### Ethics

This study was approved by the Ethics Committee of the National Center of Neurology and Psychiatry (NCNP) (B2020-032). Written informed consent was obtained from all the participants. The study was conducted in accordance with the principles of the Declaration of Helsinki. We also complied with the Ethical Guidelines for Medical and Health Research Involving Human Participants established by the Ministry of Education, Culture, Sports, Science, and Technology, the Ministry of Health, Labour and Welfare, and the Ministry of Economy, Trade, and Industry.

DNP and TRC participated in this study as co-research institutions. They explained to researchers how to use e-libraries and provided advice on the book content from the perspective of e-library experts. They also negotiated with publishers to purchase commercial e-books for the e-library. DNP and TRC were not involved in the analysis, which was conducted independently by NCNP researchers; therefore, the research results were not distorted in favour of the companies concerned.

## Results

Ninety-one participants from nine facilities participated in the study. The total number of lending e-books was 3429. The NDC code category with the most users of e-books was seven (the arts and fine arts) and two (general history). The category with the most e-books borrowed was five (technology, engineering) followed by two (Fig. 1).

Table 1 shows the demographic information. Regarding psychiatric disorders, 89.0% of participants had F2 (schizophrenia, schizotypal, and delusional disorders). The average and median number of logins were 48.3 and 20.0, respectively. The mean number of books borrowed was 37.7 (median 9.0). The mean full IQ score for the ‘over 20 times login’ group was significantly higher than that for the ‘less than 20 times login’ group (89.4 (SD 16.1) vs. 80.2 (SD 12.8), *p* < 0.006). The prescription rate of clozapine for the ‘less than 20 times login’ group was 44.4%, significantly higher than the 19.6% rate for the ‘over 20 times login’ group. The rate of antipsychotic polypharmacy (APP) tended to be lower in the ‘less than 20 times login’ group (26.1% vs. 8.8%, *p* < 0.052).

**Table 1:**
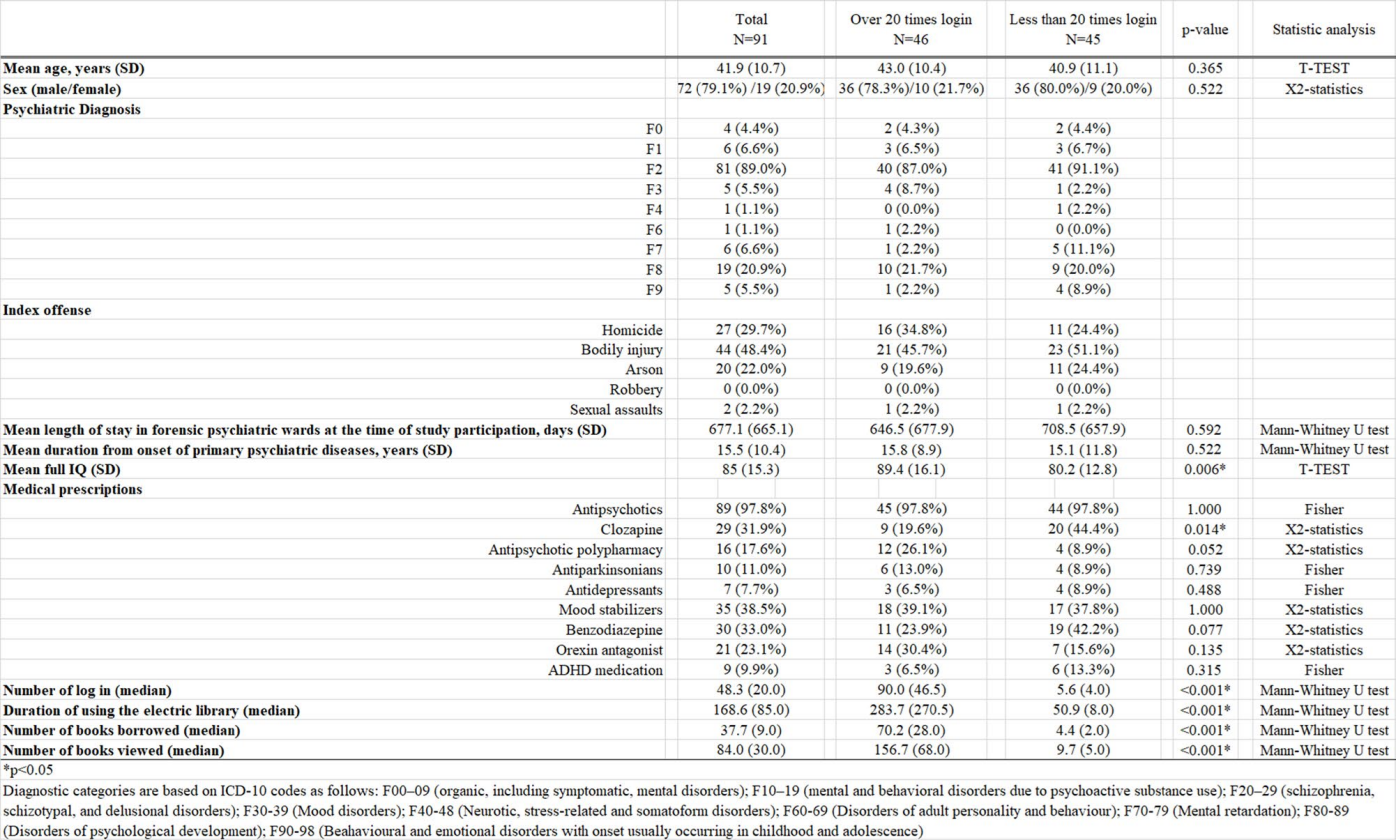
Demographic information

Eighty percent of the participants had a habit of reading books at least once a month, although over half had not used public libraries in the year before hospitalisation (Table 2).

**Table 2:**
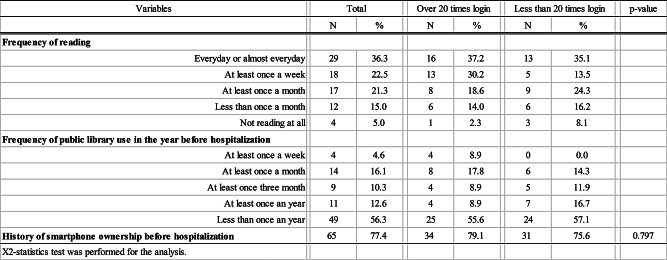
Reading habits and history of ICT equipment ownership

Table 3 shows the results of the psychological tests conducted at the start of using the e-library. The median standardised GSES score was 47.0, with no significant difference between the two groups. Among the SF-8 items, BP and MH scores were significantly higher in the ‘over 20 times login’ group. Among the Brief-COPE items, the substance use score was significantly lower in that same group. No significant differences between the two groups were measured on any of the items in the BCIS.

**Table 3:**
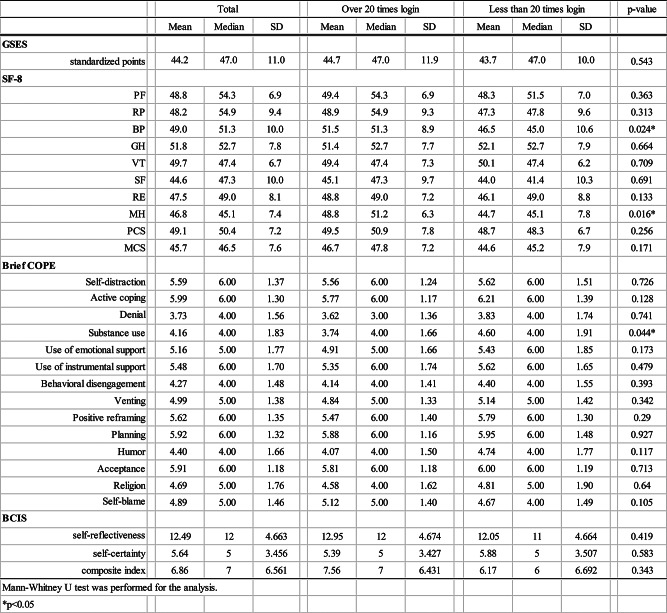
The results of psychological tests at the start of this study

Table 4 presents the change in scores over time for each psychological test item. When changes over time in the ‘over 20 times login’ group was compared with the baseline, the scores in the Brief COPE for venting at 12 weeks and religion at 26 weeks were significantly lower. Additionally, a trend towards lower scores occurred for self-distraction at 26 weeks and self-blame at 12 weeks (*p *< 0.1).

**Table 4:**
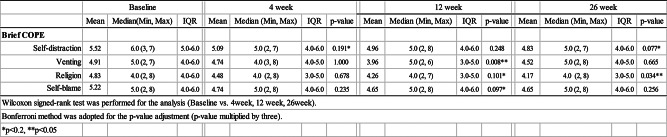
Changes in the results of psychological tests among participants with over 20 times login, listwise deletion, *n* = 23, *p* < 0.2

## Discussion

In this study, we established an e-library for forensic psychiatric inpatients that revealed its usage among these patients. We also explored which patient groups are likely to have a greater demand for the e-library.

### Demand for the e-library

According to a 2023 survey on public electronic libraries in Japan, the e-library registration rate of the population served by each public library was less than 10% at 112 facilities, 10–19% at 36 facilities, 20–29% at 22 facilities, and 30–49% at 36 facilities, while 23 facilities had a rate of 50% or higher. The remaining 96 facilities were categorised as ‘other’, ‘unknown’, or ‘no response’ [32].

Nationwide, 35 facilities with 856 beds have forensic psychiatric wards. Of these, the patients on the wards of nine facilities with 289 beds were the participants in this study. The average length of stay for forensic psychiatric inpatients is approximately three years in Japan [7]. As the research period for this study was 37 months (from February 2021 to March 2024), it can be estimated that inpatients rotated once during the study period. Therefore, the number of inpatients at participating research facilities is estimated to be approximately 600. Even taking into account differences in length of stay between facilities, the number is unlikely to exceed 1,000. Ninety-one inpatients participated in the study. Despite the burden of cooperating with regular psychological tests, at least approximately 10% of patients wanted to use the electronic library and participated in this study. The participation rate in this study was comparable to the enrolment rate in the general population.

Among public e-libraries in Japan, 34.4% have more than 10,000 e-books, 17.9% have 5000-10,000, 40.7% have 1000–5000, and 6.9% have less than 1000 e-books [32]. Thus, the number of e-books in this study was less than that in the majority of public e-libraries (the maximum number of e-books was 1478, and the minimum number was 772). To the best of our knowledge, no national survey has been conducted to quantify public e-library lending. The public e-library of Nagareyama City (which has a population of approximately 200,000) had 1,265 e-books, and the number of e-books borrowed per year was 2,785 [33]. On the other hand, a public library in another city (Kure City) with a population of around 200,000 had a collection of 8,497 e-books, and the number of e-books borrowed per year exceeded 40,000 [34]. The total number of lending e-books was 3429 in this study. As the research period was 37 months, the mean number of e-books borrowed per year was calculated to be 1,112. Considering the ratio of the population (almost 200,000 vs. unlikely to exceed 1,000), e-books were borrowed from the e-library of this study at a higher frequency than from the e-libraries of Nagareyama City and Kure City. Considering that Kure City has seven times more e-books than Nagareyama City and 30 times more e-books borrowed, it is also possible that the number of e-books borrowed by participants in this study would have increased if the collection of e-books had been more extensive.

From the perspective of comparing e-library registration rates and the number of e-books borrowed with the general population, the results in this study suggest that there is a certain demand for e-libraries among forensic psychiatric inpatients and that e-libraries have the potential to improve access to information in forensic psychiatric wards.

The category of e-books that were borrowed most frequently (799 times) was category five (technology, engineering). This category included books on home economics and life sciences, such as cooking, food, and cleaning. Reading these books may help improve social functioning. The Global Assessment of Functioning (GAF) is a numerical scale used to assess adults’ social, occupational, and psychological functioning [35]. Reports indicate that higher GAF scores are associated with lower problem behaviours and a transition to a lower-security environment [36, 37]. Having a large number of books on home economics and life sciences in the e-library may be useful from the perspective of treating forensic inpatients.

However, this category did not have many users (*n* = 34), implying that some users borrowed books frequently. As the number of participants in this study was small, the number of books borrowed for each category was influenced by a few heavy users; therefore, the number of users for each category may be more appropriate when assessing the real needs of all patients for each category. The category with the highest number of users was category seven (the arts and fine arts). This category includes books on the arts and manga, as well as books related to leisure activities such as sports, mahjong, and shogi. As leisure activities have a positive effect on mental health [38], it was considered beneficial for both patient demand and mental health to enrich the collection of e-books related to leisure activities.

### Demographic information and reading habits

Most participants in this study had schizophrenia spectrum disorders. In the overall population of forensic psychiatric inpatients in Japan, approximately 80% of patients are diagnosed with schizophrenia [7]. The ‘over 20 times login’ group had a higher full IQ score and a lower rate of clozapine prescriptions before starting the use of the e-library. Patients with treatment-resistant schizophrenia (TRS) have been reported to have poorer cognitive and community functioning than those without TRS [39, 40]. TRS is defined as schizophrenia that fails to respond to at least two antipsychotic drugs despite adequate duration of treatment, appropriate dosage, and good medication adherence [41]. Clozapine is an antipsychotic prescribed to patients with TRS [42]. The high proportion of patients with TRS might have influenced the lower full IQ score in the ‘less than 20 times login’ group. These results may be interpreted as indicating that higher cognitive function and lower disease severity before starting the use of the e-library were associated with greater frequency of e-library use. Furthermore, these results suggest that establishing e-libraries may be more beneficial for the group with higher cognitive function than for the group with lower cognitive function. To promote the use of e-libraries and enhance their benefits of introducing of e-libraries among forensic psychiatric inpatients with low cognitive and community functioning, including those with TRS, providing support regarding how to use e-libraries and to select books according to patients’ functions and needs is advisable. The APP tended to be lower in the ‘less than 20 times login’ group. In Japan, clozapine must generally be prescribed as a single agent, resulting in an extremely low APP rate in the clozapine prescription group [43]. It is presumed that the higher clozapine prescription rate in the ‘less than 20 times login’ group contributed to the tendency toward a lower APP rate.

In the ‘over 20 times login’ group, the proportion of people who read at least once a week was high at 67.4%, and four of those had frequently used public libraries at least once a week before being admitted to the forensic psychiatric wards. One inference is that the ‘over 20 times login’ group had a higher reading and library use habit prior to the research.

### Association between psychiatric symptoms at the start of e-library use and use frequency of the e-library

The results of the psychological tests conducted at the start of e-library showed that the mean standardised score for the GSES was 44.2 and the median was 47.0. As the standardised score range in adult men and women overall is 47–52 [44], the self-efficacy of the participants in this study can be assessed as somewhat lower than that of the general population. Previous research reported a correlation between reading achievement and self-efficacy [45]. However, in this study, no significant difference in self-efficacy was observed between the ‘over 20 times login’ and the ‘less than 20 times login’ groups of the e-library. In the nationwide SF-8 general sample, the mean score for the eight items was approximately 50 [46]. In this study, the mean and median scores for VT, SF, MH, and RE were all below 50, and the mean and median scores for MCS were lower than those of the general sample. These results are similar to those for a sample group with mental illnesses, such as depression [46]. The ‘over 20 times login’ group had higher MH and BP scores on the SF-8. A higher MH score indicates better mental health, while a higher BP score indicates less physical pain [46]. There are reports indicating that mental disorders such as schizophrenia may impair reading ability [47, 48]. Chronic physical pain has been reported to have a negative association with leisure activities such as reading [49]. Better mental health and less physical pain may be positively related to e-library use. Compared to patients with schizophrenia in previous Japanese reports [31, 50], the BCIS score showed similar levels of self-reflectiveness but slightly lower levels of self-certainty, resulting in a higher composite index. The BCIS is a measure of cognitive insight [30]. The inclusion of patients without schizophrenia spectrum disorders in the study and the fact that participants received adequate medication, metacognitive training, illness education, and reflection programmes in forensic psychiatric wards, may have resulted in better composite index scores.

### The impact of e-library usage on psychiatric symptoms

To evaluate the relationship between e-library usage behaviour and changes in mental state over time, we compared and analysed the results of psychological tests at the start of the study and at 4, 12, and 26 weeks for the ‘over 20 times login’ group. However, the results must be evaluated with caution because the number of participants was small and the results of the psychological tests did not follow a normal distribution.

This study found no significant changes over time in self-efficacy and health-related quality of life. To evaluate whether participants learned coping methods through reading and whether their coping methods changed, we measured using the Brief COPE method. The venting score on the Brief COPE at 12 weeks was significantly lower than that at the start of the study, but was not significantly different at 26 weeks. Compared with baseline results, religion was significantly lower at 26 weeks (*p* < 0.05). However, as forensic psychiatric inpatients receive medication and various psychosocial treatment programmes, whether e-library usage affected the decline in religion scores was unclear.

In forensic psychiatry, cognitive insight is crucial for preventing relapse and reoffending. The BCIS was measured to investigate whether reading could improve participants’ cognitive insight. However, no significant changes were observed over time.

The present study can offer limited conclusions from the longitudinal data analysis of the psychological test results. However, the study suggests that e-library usage did not have a clear negative effect on the psychiatric symptoms within the range of the results of the psychological tests.

### Challenges in the generalisation of electronic libraries within forensic psychiatric wards

Several challenges must be addressed before introducing e-libraries in forensic psychiatric wards can become generalized. Internet access via ICT devices may lead to obtaining unreliable medical information or expose patients to news related to their own criminal activities, potentially worsening their mental state. Furthermore, inappropriate emails or social media posts may cause social harm, and it is essential to protect the personal information of both patients and staff [15]. IFLA Guidelines for Library Services stipulate that collections should reflect content accuracy, inmate ethnicity and gender composition of inmates, their cultural backgrounds, language requirements, and reading levels [4]. It also states that inmates’ opinions should be consulted when adding materials. Furthermore, the libraries should provide all materials typically found on the shelves of public libraries, and restrictions on inmates’ reading materials should be kept to a minimum. However, the Guidelines notes that materials posing a clear threat to facility security or the safety of inmates and staff, as well as materials specifically designated as excluded by the facility, may exceptionally be subject to censorship.

In this study, at many participating research facilities, security settings were implemented on ICT devices using MDM (Mobile Device Management) and web filtering software to prevent their use for purposes other than accessing the electronic library. The e-books installed in this study’s electronic library were selected and purchased from the same content pool as books provided for public electronic libraries. We purchased additional e-books requested by participants. Furthermore, we did not select e-books that clearly promote criminal tendencies or books that are clearly medically inaccurate.

Criminal justice systems and forensic mental health services vary significantly between countries [51]. In Japan, only persons with mental disorders who are exempt from imprisonment because of lack of criminal responsibility or reduced responsibility due to mental disorder are admitted to forensic psychiatric wards. Moreover, all wards are uniformly classified as medium security level. Consequently, the barriers to introducing e-libraries were relatively low. However, overseas, persons with mental disorders before prosecution or those serving prison sentences may also enter forensic psychiatric wards. These patients require stricter restrictions on information access to prevent them from leaving the wards and ward safety. Furthermore, in some countries, forensic psychiatric wards feature multiple security levels, necessitating the implementation of information access restrictions commensurate with the ward’s security level. Consequently, the level of content filtering and ICT device security settings to be considered when introducing e-libraries into forensic psychiatric wards varies significantly depending on each country’s criminal justice system and forensic mental health services. Introducing e-libraries within forensic psychiatric wards should be advanced in a manner adapted to each country’s specific circumstances, balancing risks and benefits.

### Limitations

This study has several limitations. First, due to differences in budgets, ward structures, and ward practices among facilities, there were differences in available ICT devices, time and place where ICT devices can be used, and security management methods. Therefore, it was not possible to evaluate the impact of participants’ reading environments. Second, the number of e-books in the library varied depending on the time of year; therefore, it is possible that the time of year when the patients participated had an impact on their reading behaviour. Third, this study is limited to research participants who provided informed consent. Therefore, patients who participated in the study were interested in using e-libraries and confident in their ability to access electronic libraries using ICT devices. As a result, the study sample may have high social functioning and high motivation to use e-libraries. Fourth, a majority of the participants in this study were persons with schizophrenia. As the disease structure of forensic psychiatric inpatients varies by country, the generalisability of these findings is limited. Fifth, although the e-library was set up for this study, we did not recruit control institutions and cases. Longitudinal changes in inpatients’ psychiatric symptoms in the absence of the electronic library were unknown. Consequently, it was challenging to assess the effectiveness of the e-library itself as an ‘intervention’. Therefore, the findings of this study cannot address causality. A longitudinal study incorporating a control group is desirable. Sixth, in this study, the data were divided based on the median frequency of e-library use because the sample size was relatively small and the distribution of usage frequency was highly skewed. As a results, the statistical power was limited. Future research should include a larger sample and apply continuous models and regression analysis to improve analytical robustness. Whether the bias in frequency of use is due only to the small sample size or whether selection bias, facility characteristics and other factors also contribute needs to be further investigated in future studies. Furthermore, we were unable to consider other potential factors inherent in facility characteristics, such as the state of digital infrastructure, general practice, and function. Facility characteristics should also be investigated and analysed in further studies in the future.

## Conclusion

In this study, we supplied an e-library for forensic psychiatric inpatients and the results demonstrated their demand for its use and identified factors that may be related to e-library use behaviour. The use of e-libraries developed specifically for forensic psychiatric wards using ICT equipment with appropriate security regarding the use of the Internet could contribute to an improved environment for information access without adversely affecting patients’ psychiatric symptoms. The results of this study are promising and support the feasibility of an e-library in the field of forensic psychiatry. However, there are several limitations, and caution is required when evaluating the results. Further research using a longitudinal or comparative study design is needed. In addition, qualitative feedback should be incorporated to investigate implementation barriers in diverse forensic ward settings. This will enable further examination of the impact of digital access in a safe environment on clinical and ethical outcomes.

**Appendix 1:**
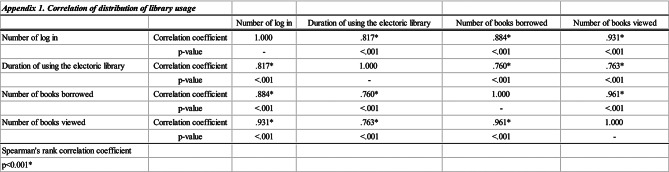
Correlation of distribution of library usage

## Data Availability

The data supporting the findings of this study are not available because consent for public data sharing was not obtained from the participants.

## Abbreviations

APP: Antipsychotic polypharmacy
BCIS: Beck Cognitive Insight Scale
BP: Bodily pain
Brief COPE: Brief-Coping Orientation to Problems Experienced
COPE: Coping Orientation to Problems Experienced
DNP: Dai Nippon Printing Co., Ltd
GH: General health
GSES: General Self-Efficacy Scale
HRQOL: Health-related quality of life
ICT: Information and communication technology
ICD-10: International Classification of Diseases, 10th edition
BCIS-J: Japanese version of the BCIS
MCS: Mental component score
MH: Mental health
NDC: Nippon Decimal Classification
PCS: Physical component score
PF: Physical functioning
RE: Role emotional
RP: Role physical
SF-8: Short Form-8 Health Survey
SF: Social functioning
SD: Standard deviation
TRC: TRC Library Service Inc
VT: Vitality
WAIS-III or IV: Wechsler Adult Intelligence Scale
